# Plasma Markers of Inflammation Linked to Clinical Progression and Decline During Preclinical AD

**DOI:** 10.3389/fnagi.2019.00229

**Published:** 2019-09-06

**Authors:** Alden L. Gross, Keenan A. Walker, Abhay R. Moghekar, Corinne Pettigrew, Anja Soldan, Marilyn S. Albert, Jeremy D. Walston

**Affiliations:** ^1^Department of Epidemiology, Johns Hopkins Bloomberg School of Public Health, Baltimore, MD, United States; ^2^Department of Neurology, Johns Hopkins School of Medicine, Baltimore, MD, United States; ^3^Division of Geriatric Medicine and Gerontology, Johns Hopkins School of Medicine, Baltimore, MD, United States

**Keywords:** preclinical AD, inflammation, cytokines, cognitive decline, biomarkers, TNFR1

## Abstract

**Objective:**

To examine the prospective association between blood biomarkers of immune functioning (i.e., innate immune activation, adaptive immunity, and inflammation) and subsequent cognitive decline and clinical progression to mild cognitive impairment (MCI) in cognitively normal individuals.

**Methods:**

The BIOCARD study is an observational cohort study of *N* = 191 initially cognitively healthy participants (mean age 65.2 years). Blood plasma samples were assayed for markers of chronic inflammation (TNFR1, IL-6), adaptive immunity (CD25), and innate immune activation (CD14 and CD163). Participants were followed annually for ongoing clinical assessment and cognitive testing for up to 7.3 years. Primary study outcomes were progression to MCI and cognitive change over time, as measured by a global factor score encompassing multiple cognitive domains.

**Results:**

Higher levels of plasma TNFR1 were associated with greater risk of progression from normal cognition to MCI (HR: 3.27; 95% confidence interval, CI: 1.27, 8.40). Elevated levels of TNFR1 were also associated with steeper rate of cognitive decline on follow-up but not with baseline cognitive performance. Baseline IL-6 levels and markers of innate and adaptive immune activation showed no relationship with MCI risk or cognitive decline.

**Conclusion:**

Inflammation, mediated by TNF signaling, may play a selective role in the early phase of AD. Accordingly, plasma TNFR1 may facilitate improved prediction of disease progression for individuals in the preclinical stage of AD.

## Introduction

Complex and chronic inflammatory responses are evident in the brains of patients with Alzheimer’s disease (AD) dementia. Immune-related cells including astrocytes and microglia cluster around αβ plaques (Rozemuller et al., 1986; Itagaki et al., 1989). These cells are thought to release inflammatory cytokines, such as tumor necrosis factor-alpha (TNF-α), which further activate neuroinflammation (Tuppo and Arias, 2005; Holmes et al., 2009; Heneka et al., 2015; Wang et al., 2015; Saresella et al., 2016) as well as further cytokine production (Rubio-Perez and Morillas-Ruiz, 2012). Additionally, recent findings related to genetic risk factors for AD, such as TREM2 expressed on microglia (Jin et al., 2014), have re-emphasized the importance of inflammation in the evolution of AD (Chiappelli et al., 2006; Van Cauwenberghe et al., 2016).

Several previous studies have examined the relationship between blood measures of inflammation and cognition, based on either cross-sectional or limited longitudinal data. The most common finding has been that IL-6, measured in community-dwelling populations with a range of cognition at first assessment, is associated with subsequent cognitive decline (e.g., Weaver et al., 2002; Adriaensen et al., 2014; Singh-Manoux et al., 2014). However, few studies have evaluated the relationship between cognition and a range of markers of immune functioning in addition to chronic inflammation, including innate immune activation and adaptive immunity. To develop a more complete understanding of how distinct aspects of immune function relate to cognitive decline, and to gain insight into how discrete immune pathways may play a role in the pathophysiology of AD, it is important to consider a range of inflammatory markers. Consideration of multiple markers could provide more effective diagnostic and prognostic measurement strategies and future targets for intervention strategies. Thus, in this study, we measured circulating markers of chronic inflammation (TNFR1, IL-6), markers of innate immune activation (CD14 and CD163), and an adaptive immunity marker (CD25, a marker of T-cell activity) to determine whether these measures are associated with cognitive decline and incidence of MCI. These immune system measures were chosen in part because they have not been extensively studied in populations of older adults with respect to cognitive decline, and because their domain specificity may provide unique insights into potential biological connections underlying specific immune pathways and cognitive decline.

For markers of chronic inflammation, we chose interleukin-6, a commonly measured cytokine often associated with adverse health outcomes in older adults, and tumor necrosis factor alpha receptor one (TNFR1). The ligand that binds to TNFR1 is TNF-α, an early phase inflammatory cytokine that has been hypothesized to be a key mediator of AD pathology based on studies in mice (McAlpine et al., 2009) and humans (Wang et al., 2018). Indeed, elevated blood levels of TNF-α are associated cross-sectionally with cognitive impairment in individuals with AD dementia (Alvarez et al., 1996; Holmes et al., 2009) and steeper longitudinal cognitive decline over the short term in older adults with a range of cognitive function at first assessment (Weaver et al., 2002; Adriaensen et al., 2014). TNFR1 is an easily measureable and robust marker of chronic inflammatory pathway activation, and has been previously associated with mortality and other adverse health outcomes in older adults (Varadhan et al., 2014).

While systemic inflammation has been previously implicated in the development and progression of AD, there is only limited evidence for a potential role of innate immune activation. CD14 and CD163 are cell surface makers from cells of the monocyte/macrophage lineage. These cells play an important role in moderating and sustaining inflammatory reactions and are generally elevated in chronic infections or injuries. Previous research suggests higher soluble CD14 levels have been found in individuals with dementia compared to cognitively normal individuals (Yin et al., 2009). Additionally, elevated levels of soluble CD163 have been associated with age-related diseases including atherosclerosis (Aristoteli et al., 2006) and rheumatoid arthritis (Greisen et al., 2011), which may themselves influence dementia risk.

Adaptive immunity is known to be altered in older age. T cell subsets in particular have been shown to regulate aspects of amyloid pathology in AD transgenic rodent models (Cao and Zheng, 2018). However, whether blood markers of T- and B-cell activity, such as CD25/IL-2R (Leipe et al., 2005), are associated with cognitive decline during the preclinical phase of AD remains unknown. To our knowledge, the association of CD25 levels with risk of cognitive decline and early impairment in humans has yet to be evaluated.

Building on these prior studies, we designed a study to identify whether the activation of discrete inflammatory and immune pathways influence cognitive decline and clinical progression in a well-characterized cohort of adults that has been followed longitudinally for many years. We hypothesized that early changes in plasma levels of inflammatory markers are associated with cognitive decline and progression to MCI among cognitively normal people.

## Materials and Methods

### Participants

We used data from the Biomarkers of Cognitive Decline among Normal Individuals (BIOCARD) study. The BIOCARD study began in 1995 at the Geriatric Psychiatry branch of the Intramural Program of the National Institute of Mental Health. Data were collected until 2005 in the initial phase of the study. The study initially included *N* = 349 older adults with normal cognition, most of whom were middle-aged at enrollment (mean age 57 years) and by design had a first-degree relative with dementia. The study was designed to follow cognitively normal individuals to identify early cognitive, imaging, and biospecimen markers that predict progression to mild cognitive impairment (MCI), under the premise that AD pathophysiologic processes begin prior to the onset of clinical symptoms.

Data collection was reestablished in 2009 by researchers at the Johns Hopkins School of Medicine (Albert et al., 2014), which involved reinitiating annual clinical assessments and cognitive testing, and collection of blood specimens. The blood plasma samples assayed in this project are from visits between September 2009 and November 2011, which represents participants’ first visit at Johns Hopkins. Assays were completed in 191 cognitively normal participants, which constituted the sample used for the present study.

### Standard Protocol Approvals, Registrations, and Patient Consents

All participants provided written informed consent, and the study was approved by The Johns Hopkins University Institutional Review Board.

### Immune Function Measures

We assayed plasma samples for markers of chronic innate immune system activity (TNFR1, IL-6), innate immune activation (CD14, CD163), and adaptive immunity (CD25, a marker of T-cell activity). These selected markers are each typical hallmarks of the processes they are intended to reflect in this study. All samples were measured in duplicate using human IL-6, CD163, CD25, and soluble CD14 Quantikine ELISA Kits from Fisher Scientific, Waltham, MA, United States. Plates were read with MicroPlate Reader 680 from BioRad. Soluble TNFR1 was measured using a Mesoscale chemiluminiscent singleplex assay (Varadhan et al., 2014). Because of variability in TNF-α measurement from stored samples, we evaluated soluble levels of its receptor, TNFR1, as a proxy measure of TNF-α; they are highly correlated (Griffin et al., 1995). The coefficients of variation for assayed measures of IL-6, TNFR1, CD14, CD25, and CD163 in this sample were respectively 1.74, 0.45, 0.28, 0.81, and 0.49.

### Neuropsychological Testing

Cognitive assessments in BIOCARD are extensive and span multiple domains including episodic memory, executive function, language, spatial ability, attention, and psychomotor speed (Albert et al., 2014). We used data from tests that have shown associations with progression from normal cognition to MCI (Table 1). The Logical Memory (immediate and delayed recall) and Paired Associate (immediate recall) subtests from the Wechsler Memory Scale – Revised (Wechsler, 1987) assess verbal episodic memory. The Digit Symbol Substitution test of the Wechsler Adult Intelligence Scale – Revised (WAIS-R) involves matching pairs of digits to symbols (Wechsler, 1981), and measures processing speed, incidental memory and executive function. The Block Design subtest, also from the WAIS-R, is a test of motor and visuospatial ability in which participants arrange blocks into patterns.

**TABLE 1:** Descriptive characteristics of the sample (*N* = 191).

**Variable**	**Full sample (*N* = 191)**	**Remained normal (*N* = 168)**	**Progressed to MCI (*N* = 23)**	***p*-value for difference**	**Range**
					
	**Mean (SD) or *N* (%)**	**Mean (SD) or *N* (%)**	**Mean (SD) or *N* (%)**		
Age, years	65.2 (8.9)	64.2 (8.7)	72.4 (6.9)	<0.001	28, 87
Education, years	17.4 (2.2)	17.4 (2.1)	16.9 (2.4)	0.276	12, 20
Female sex, *N* (%)	119 (62.3)	110 (65.5)	9 (39.1)	0.014	
Vascular risk score	1.3 (1.1)	1.3 (1.1)	1.8 (1.1)	0.023	0, 4
Years of follow-up in study	5.8 (1.1)	5.8 (1.1)	5.8 (1.1)	0.931	1, 7.3
Biomarkers					
IL-6, pg/ml	2.4 (4.3)	2.5 (4.5)	1.6 (0.8)	0.368	0.4, 32.3
TNF a-R-1, pg/ml	1026.8 (497.5)	969.3 (285.6)	1446.6 (1144.0)	<0.001	503.8, 6423.2
CD14, ng/ml	1382.9 (411.2)	1369.9 (392.8)	1477.6 (527.3)	0.24	730.3, 3440.0
CD25, ng/ml	1014.9 (933.9)	966.8 (772.6)	1366.1 (1689.8)	0.054	407.7, 9460.0
CD163, ng/ml	461.3 (226.5)	455.3 (211.9)	505.2 (316.0)	0.323	123.4, 1548.8
Cognitive tests					
General cognitive factor	−0.1(0.8)	0.0 (0.8)	−0.8(0.6)	<0.001	−2.7, 1.6
Logical memory, Immediate recall	16.2 (3.2)	16.5 (3.1)	14.2 (2.8)	0.001	8.0, 24.0
Logical memory, Delayed recall	15.4 (3.4)	15.7 (3.4)	13.0 (2.3)	<0.001	5.0, 24.0
Paired associates learning	19.5 (3.1)	19.8 (3.0)	17.5 (3.1)	<0.001	7.0, 24.0
Digit symbol substitution test	56.1 (11.5)	57.4 (11.4)	46.6 (7.2)	<0.001	29.0, 87.0
Block design	33.3 (8.9)	33.9 (8.8)	28.7 (9.1)	0.008	11.0, 51.0

*Statistics for raw biomarker concentrations are provided in this table. Log-transformed values were used in parametric models.*

A factor score representing general cognitive performance at each study visit using longitudinal confirmatory factor analysis methods of the tests above was constructed (Gross et al., 2017). Briefly, factor scores from two-parameter logistic item response models of the cognitive test scores were estimated (Muthen and Muthen, 1998–2018). Item-level fit of data in the models was evaluated using normalized residuals (Bollen, 1989).

### Clinical Progression Determination

The BIOCARD study team reviews clinical and cognitive characteristics of participants after each annual visit and generates a consensus clinical diagnosis (Albert et al., 2014). A consensus diagnosis for each subject at each visit is based on cognitive test performance, medical, neurologic and psychiatric assessments, and a semi-structured interview based on the Clinical Dementia Rating (CDR) scale (Morris, 1993), given to the participant and informant. Diagnostic procedures conform to recommendations from the NIA-AA workgroup reports for the diagnosis of MCI (Albert et al., 2011) and dementia due to AD (McKhann et al., 2011). The medical, neurologic, and psychiatric status of each participant is evaluated based on clinical data, reports of cognitive decline are based on self-report and informant reports, and objective cognitive decline is identifiable from the BIOCARD neuropsychological battery. Likely etiology (e.g., Alzheimer’s, vascular, etc.) was assigned based on medical, neurologic, and psychiatric information available, and more than one etiology could be selected. The estimated age of onset of clinical symptoms was based on the subject and informant reports derived from the CDR interview (see the Supplementary Appendix e-1 for details of these diagnostic procedures).

### Covariates

All analyses are adjusted for age at immune biomarker ascertainment, years of education, sex, and vascular risk factors using a previously published summary composite based on the presence or absence of five risk factors, including smoking status, hypertension, hypercholesterolemia, diabetes, and body mass index (BMI) ≥ 30 (Gottesman et al., 2017). In regressions, age was discretized approximately into quartiles (28–60 years; >60–65 years; >65–75 years, >75–93 years) to accommodate potential non-linear associations between age and cognitive outcomes.

### Analysis Plan

We examined distributions of each immune marker to assess their shape and the presence of outliers. We calculated logarithms of biomarkers to approximate normality. To model the time to progression to incident MCI as a function of each biomarker separately, we used Cox proportional hazards models with the Efron method for handling ties (Hertz-Picciotto and Rockhill, 1997). Participants contributed time from the age of biomarker ascertainment to estimated age of onset of the symptoms of MCI or the age at the last date of study follow-up for those who remained normal. To assess the relationship between cognitive trajectories of general cognitive performance with each biomarker, we used latent growth curve modeling with age as the timescale and random effects for person and time. In a secondary analysis, we modeled change in each component test. Participants contributed time to the longitudinal analyses after their biomarker ascertainment. All models were adjusted for age, sex, years of education, and vascular risk score to address potential confounding. Missing covariate data were negligible. For survival analyses, time to event (progression or censoring) were available for the entire sample. Latent growth curve models with a robust maximum likelihood estimator appropriately treat missing data in dependent variables, including loss to follow-up, as missing at random conditional on variables in the model.

### Data Availability

Anonymized study data from the BIOCARD study, including data not published in this article, are available upon request to any qualified researcher for purposes of replicating the results.

## Results

### Descriptive Statistics

As shown in Table 1, the 191 participants in this study were aged 65.2 years on average when the plasma samples were collected. Participants were highly educated (17.4 years of education on average) and were 62% female. They were followed for an average of 5.8 years and up to 7.3 years. Means and ranges for immune biomarkers and cognitive tests are in Table 1. At baseline, no participants were missing data on any variable in Table 1.

### Survival Analysis

**FIGURE 1:**
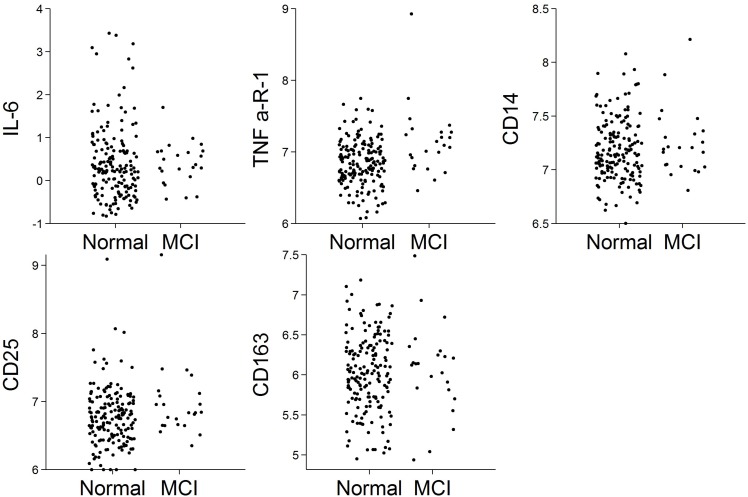
Scatterplots of biomarkers by follow-up diagnosis (*N* = 191). Biomarker values were log-transformed.

**FIGURE 2:**
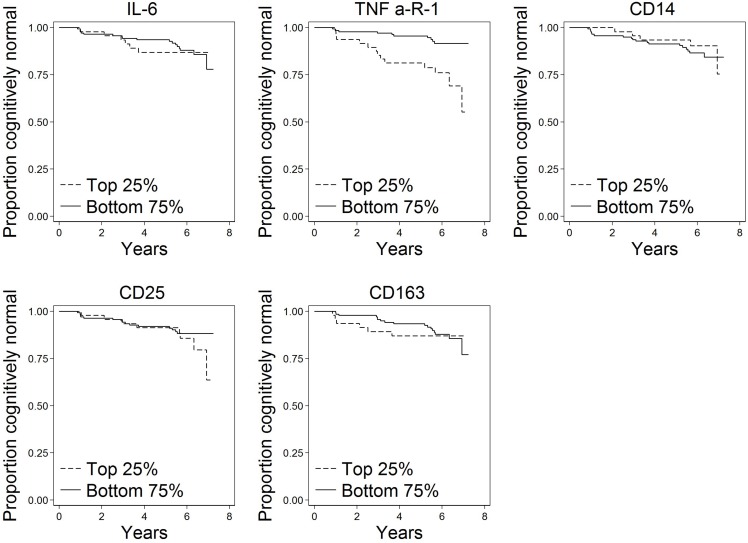
Kaplan–Meier plots of time to onset of MCI by top quartile of each biomarker (*N* = 191). These plots show time to progression from normal cognition to onset of MCI by biomarker level. A progressor is a participant who was cognitively normal at baseline and developed clinical symptoms of MCI during follow-up as determined by a standardized clinical adjudication procedure (described in the section “Materials and Methods” and Supplementary Appendix e-1).

Of the *N* = 191 cognitively normal participants who provided blood specimens for analyses, *N* = 23 (12%) progressed to MCI over the follow-up. Participants contributed 1,036 person-years to the survival analysis. Figure 1 shows scatterplots by follow-up diagnosis (remained normal, progressed to MCI) for each biomarker. Levels of TNFR1 appear elevated among participants who eventually progressed. Kaplan–Meier plots in Figure 2 suggest that levels of TNFR1 in the top quartile of the distribution are associated with elevated risk of progression to MCI.

Hazard ratios (HR) for each immune biomarker are in Table 2, all of which are adjusted for age, sex, years of education, and vascular risk score. Hazard ratios represent risk of clinical symptoms of MCI onset per log unit difference in biomarker level. Elevated levels of TNFR1 were associated with greater risk of progression to a diagnosis of MCI (HR: 3.27; 95% confidence interval, CI: 1.27, 8.40). The magnitude of this association of TNFR1 with the hazard of progression to MCI is comparable to the effect of being nearly 3 years older at study entry on the risk of progression to MCI, given that the hazard ratio for age, adjusting for other covariates, is 1.12. No other biomarkers were associated with progression to MCI.

**TABLE 2:** Hazard ratios for time to progression to MCI (*N* = 191).

**Biomarker predictor**	**Hazard ratio**	**95% Confidence interval**	***p*-value**
IL-6	0.57	(0.24, 1.35)	0.2
TNFR1	3.27	(1.27, 8.40)	0.01
CD14	2.29	(0.49, 10.75)	0.29
CD25	1.65	(0.79, 3.48)	0.19
CD163	0.86	(0.34, 2.19)	0.76

*Hazard ratios represent risk of MCI onset per log unit difference in biomarker level. Values greater than 1 suggest greater levels of the biomarker are related to increased risk of MCI. Models are adjusted for age, sex, years of education, and vascular risk factors.*

Of the *N* = 23 progressors in our analysis, the clinically adjudicated most common etiology involved AD in 14 cases, of which eight included a mixture of AD and vascular contributions to cognitive impairment. There were five cases where vascular pathology was presumed to be the primary etiology (the remaining four cases included possible fronto-temporal dementia and cognitive dysfunction from medications). We repeated the survival analyses for those with AD and vascular contributions to cognitive impairment (*n* = 19), using an indicator variable for possible combinations (e.g., AD but not vascular, AD and vascular, vascular but not AD, etc.). Associations of TNFR1 remained significant for the cases with likely AD pathology only (HR: 3.76; 95% CI: 1.09, 13.00), vascular pathology only (HR: 5.27; 95% CI: 1.63, 17.03), and both (HR: 7.79; 95% CI: 1.35, 44.89).

Noting a higher prevalence of progressors to MCI among males (19%) than females (8%), we subsequently tested for sex differences in the associations of each immune biomarker with progression to MCI in a sensitivity analysis. Hazard ratios did not differ by sex for four of biomarkers (*p* = 0.3). An exception was CD163, which was not statistically significantly associated with risk of progression in men (HR: 2.39; 95% CI: 0.78, 7.31) but was strongly protective among women (HR: 0.047; 95% CI: 0.0055, 0.40) (*p*-value for effect modification by sex < 0.001). In another sensitivity analysis, we tested whether APOE ε4 status modified the associations of biomarkers with risk of progression. We found no such associations.

### Cognitive Trajectory Analysis

Elevated levels of TNFR1 were associated with steeper general cognitive decline on follow-up (beta = -0.012 SD units per log-transformed unit difference in TNFR1, 95% CI: -0.02, -0.0002), but not with differences in the baseline level of cognitive performance (Table 3). In secondary analyses, we examined the relationship between biomarker levels and cognitive trajectories separately for each of the cognitive measures included in the general factor score (Supplementary Table e-1). Associations between elevated levels of TNFR1 with cognitive decline were strongest for delayed recall of the Logical Memory test. Elevated levels of TNFR1 were also marginally associated with steeper decline in the digit symbol substitution test. Additionally, higher levels of CD25 were associated with steeper declines in performance on the digit symbol substitution test (Supplementary Table e-1). There were no differences in the associations of biomarkers with cognitive decline by sex or APOE ε4 status.

**TABLE 3:** Associations of biomarkers with trajectories of global cognitive performance (*N* = 191).

**Biomarker predictor**	**Association with cognitive slope**			**Association with cognitive level**		
		
	**Beta**	**95% Confidence interval**	***p*-value**	**Beta**	**95% Confidence interval**	***p*-value**
IL-6	−0.002	(−0.01, 0.01)	0.689	0.17	(−0.46, 0.80)	0.60
TNF a-R-1	−0.012	(−0.02, −0.0002)	0.046	0.80	(−0.07, 1.67)	0.07
CD14	−0.003	(−0.02, 0.01)	0.668	0.20	(−0.71, 1.11)	0.67
CD25	−0.006	(−0.02, 0.01)	0.391	0.43	(−0.47, 1.33)	0.35
CD163	−0.009	(−0.02, 0.00)	0.134	0.60	(−0.16, 1.36)	0.12

*Beta coefficients for the cognitive slope represent the mean difference in rate of cognitive change per log-transformed unit difference in biomarker levels. Beta coefficients for the cognitive level refer to the mean difference in baseline level of cognitive performance per log-transformed unit difference in biomarker levels. To facilitate comparison of coefficients across tests, cognitive tests were *z*-standardized to have a mean of 0 and variance of 1 prior to regression. A different model was estimated for each biomarker. Models are adjusted for age, sex, years of education, and vascular risk factors.*

## Discussion

The current study, which examined the association between circulating markers of immune function and clinical progression among a group of cognitively normal participants, found that elevated levels of TNFR1 are associated with greater risk of progression to incident MCI as well as steeper cognitive decline. However, TNFR1 and other immune markers were unrelated to baseline levels of cognition. This finding is consistent with the hypothesis that inflammatory changes, mediated through TNF-α signaling, may occur early in AD, prior to the emergence of clinically significant symptoms of MCI. Importantly, we found no significant association of markers of innate immunity (i.e., CD14/CD163) and adaptive immunity (CD25/sIL-2Ra) with clinical progression or cognitive decline in the full sample. These findings suggest that in the preclinical phase of AD, measures of chronic systemic inflammation may be the most relevant for prognostication, as well as for understanding the role of inflammation during this early phase of AD.

Our findings are consistent with and extend previous research in subjects in later phases of disease. For example, TNFR1 has been shown to be associated with risk of clinical progression among people with MCI (Buchhave et al., 2010; Diniz et al., 2010). Also consistent with our findings, one review of 118 mostly cross-sectional studies noted that the strongest evidence for upregulation of TNF-α is from studies comprised of people with mild AD dementia (Brosseron et al., 2014).

TNFR1 affects several pathophysiologic pathways, including beta-amyloid (AB) metabolism that may offer insights into potential pathological processes by which chronic innate immune system activity affects risk of progression from normal cognition to MCI. In animal models, TNFR1 is associated with amyloidosis and APP processing (He et al., 2007; Paouri et al., 2017). Additionally, TNFR1 is a strong activator of apoptosis and triggers necroptosis. Recent studies in animal models have demonstrated that higher TNFR1 levels are associated with morphological damage in choroid plexus epithelial cells along the blood-brain barrier; removal thereof is associated with less degradation of the blood-brain barrier, less neuroinflammation, and ultimately less amyloidosis (Steeland et al., 2018). That we identified associations of elevated TNFR1 levels with steeper cognitive decline, but not with cross-sectional cognitive performance, underscores the potential importance of this pathological process early on in AD pathophysiology when AB accumulation is accelerating. Because both tau and amyloid can accumulate in normal individuals during mid-life (e.g., Pletnikova et al., 2018), progression from normal to MCI in this study might be driven by the acceleration of synaptic dysfunction resulting from these combined pathological processes.

IL-6 and markers of innate and adaptive immune activation showed no relationship with clinical progression or cognitive decline in the full sample in the present study. Given that both TNFR1 and IL-6 are commonly regarded as pro-inflammatory cytokines, their discordant association with cognitive decline was unexpected and suggests, perhaps, that these two cytokines relate differentially to progression of disease during the preclinical phase of AD. While there are previous reports of elevated IL-6 levels in individuals with AD (Brosseron et al., 2014), several studies of non-demented participants have found no association of IL-6 with cognitive decline and clinical progression (Metti et al., 2014a, b). Like IL-6, levels of CD40 were also not altered in cognitively normal individuals who subsequently progressed to MCI in the current study. However, previous studies have consistently reported elevated CD40 in patients with AD dementia (Ait-ghezala et al., 2008; Doecke et al., 2012). Thus, while IL-6 and CD40 appear to be elevated in the context of AD, these changes may not occur until later in the disease course than TNFR1, after clinically significant symptoms have emerged.

Sex differences in AD biomarkers is an active area of research (Deming et al., 2018). CD163 is a scavenger receptor, expressed on monocytes and macrophages, important for homeostatic regulation of immune function (Onofre et al., 2009). We selected this biomarker because of its associations with atherosclerosis and rheumatoid arthritis (and thus a plausible relationship with cognitive decline), but there are sex differences in the influence of various risk factors with these diseases. There is some evidence that estrogen blunts macrophage activation (Toniolo et al., 2015). It is thus plausible that blunted levels of CD163 in women confer a protective effect on risk of cognitive deterioration. We note, however, that there were no sex differences in the association of CD163 with cognitive decline. Thus, replication of a sex-specific effect of CD163 is especially important before drawing conclusions.

This study has several strengths. We leveraged extensive follow-up of a cognitively normal cohort, with well-characterized cognitive performance and clinical outcomes. Most prior studies of chronic inflammation in this area are cross-sectional, or have insufficient longitudinal follow-up to characterize clinical progression, thus limiting our understanding of the potential role of chronic inflammation in pathological processes during early stages of AD. A second strength is that we were able to examine multiple domains of immune function; previous studies have focused on a more limited number of markers, generally inflammatory cytokines.

Several study limitations should be noted. Chronic systemic inflammation is more likely than short-term inflammation to play a role in AD pathogenesis (Cunningham et al., 2005). Biomarkers levels in the present study were assayed at a single occasion, thus capturing a single snapshot that may be representative of short term rather than chronic inflammatory pathway activation. Also, cross-sectional biomarker measurement likely contains random measurement error that likely would act to increase variability or noise around true associations. That we detected relationships for TNFR1 despite this limitation speaks to the magnitude of the relationship. A further limitation is that only 12% of the sample progressed to MCI over an average of 5.8 years of follow-up in the study. The relatively modest number of progressors limits the power to detect associations; with a larger number of progressors, additional associations may be identified. Follow-up time was not differential in this study by progression status, thus results are unlikely to be systematically biased. Related to this limitation, additional follow-up would improve power and could reveal additional associations. We note in Figure 2 a separation in risk of MCI progression between the top and bottom quartiles of CD25 apparent at the end of follow-up; although sample size is thin in this range, additional follow-up could reveal an effect of CD25 later than TNFR1. Finally, our study of inflammatory domains was not comprehensive enough to capture all potential markers and mechanisms. Although the biomarkers we selected are typical of the processes they are intended to reflect in this study, we evaluated just a few markers reflecting each process. Indeed, many other potential markers of inflammation could have been chosen for this study based on prior associations of tissue findings. For example, CRP has been found in neurofibrillary tangles and neuritic plaques in brains of people with AD (Duong et al., 1997), and soluble CD40 has been shown to be elevated in plasma of people with AD (Mocali et al., 2004; Ait-ghezala et al., 2008; Doecke et al., 2012) and predictive of progression to AD (Buchhave et al., 2009).

In conclusion, our results demonstrate that TNFR1, measured before clinically apparent symptoms, may facilitate improved prediction of which cognitively normal individuals will progress to MCI and dementia and highlight potential pathological processes underlying progression to prodromal AD. This strong longitudinal relationship suggests an important role for chronic inflammatory pathway activation in the progression of cognitive decline. Even if TNFR1 is a non-causal marker of individuals who are destined to develop MCI due to AD, evidence of early inflammatory pathway activation specific to this pathway is an important finding. Future research is needed to evaluate a potential clinical role of this and other biomarkers in the identification of those at higher risk for future cognitive impairment and decline. Such studies would also provide important biological insights that could be targeted in future treatment and prevention strategies.

## Data Availability

The datasets generated for this study are available on request to the corresponding author.

## Author Contributions

AG, KW, AM, CP, AS, MA, and JW drafted and revised the manuscript for content. AG, JW, and MA conceived or designed the study and obtained funding. AG contributed to the statistical analysis. MA and JW supervised or coordinated the study.

## Conflict of Interest Statement

MA is an advisor to Eli Lilly. The remaining authors declare that the research was conducted in the absence of any commercial or financial relationships that could be construed as a potential conflict of interest.
