# Supplementary material for: Plasma Markers of Inflammation Linked to Clinical Progression and Decline During Preclinical AD
Source: Front Aging Neurosci. 2019 Sep 6;11:229. doi: 10.3389/fnagi.2019.00229 (PMC6742958; doi:10.3389/fnagi.2019.00229)
Supplement: Supplementary file 1 [file Table_1.DOCX]

Table e-1. Associations of biomarkers with trajectories of cognitive performance, with individual cognitive tests serving as the dependent variable (N=191)

| Biomarker predictor |  | Association with cognitive slope | | |  | Association with cognitive level | | |
| --- | --- | --- | --- | --- | --- | --- | --- | --- |
|  | Cognitive outcome | Beta | 95% Confidence interval | p-value |  | Beta | 95% Confidence interval | p-value |
| IL-6 |  |  |  |  |  |  |  |  |
|  | Logical Memory, Immediate Recall | 0.02 | (-0.03, 0.06) | 0.53 |  | -0.96 | (-4.26, 2.35) | 0.57 |
|  | Logical Memory, Delayed Recall | 0.00 | (-0.06, 0.05) | 0.91 |  | 0.31 | (-3.35, 3.97) | 0.87 |
|  | Paired associates learning | 0.01 | (-0.03, 0.05) | 0.65 |  | -0.46 | (-3.02, 2.10) | 0.73 |
|  | Digit Symbol Substitution Test | 0.03 | (-0.15, 0.10) | 0.67 |  | 2.63 | (-6.14, 11.39) | 0.56 |
|  | Block Design | 0.01 | (-0.09, 0.07) | 0.85 |  | 1.00 | (-4.30, 6.30) | 0.71 |
| TNFR1 |  |  |  |  |  |  |  |  |
|  | Logical Memory, Immediate Recall | 0.03 | (-0.08, 0.03) | 0.37 |  | 1.86 | (-2.09, 5.81) | 0.36 |
|  | Logical Memory, Delayed Recall | 0.07 | (-0.13, -0.00) | 0.04 |  | 4.49 | (0.16, 8.82) | 0.04 |
|  | Paired associates learning | 0.00 | (-0.05, 0.06) | 0.89 |  | -0.50 | (-4.37, 3.38) | 0.80 |
|  | Digit Symbol Substitution Test | 0.19 | (-0.38, 0.01) | 0.06 |  | 12.54 | (-1.18, 26.27) | 0.07 |
|  | Block Design | 0.05 | (-0.13, 0.03) | 0.25 |  | 2.28 | (-3.80, 8.36) | 0.46 |
| CD14 |  |  |  |  |  |  |  |  |
|  | Logical Memory, Immediate Recall | 0.01 | (-0.04, 0.06) | 0.71 |  | -0.87 | (-4.54, 2.81) | 0.64 |
|  | Logical Memory, Delayed Recall | 0.01 | (-0.07, 0.06) | 0.86 |  | 0.21 | (-4.33, 4.76) | 0.93 |
|  | Paired associates learning | 0.00 | (-0.05, 0.06) | 0.97 |  | -0.18 | (-3.87, 3.50) | 0.92 |
|  | Digit Symbol Substitution Test | 0.12 | (-0.27, 0.03) | 0.12 |  | 8.70 | (-1.28, 18.69) | 0.09 |
|  | Block Design | 0.00 | (-0.09, 0.09) | 0.98 |  | -0.48 | (-6.59, 5.64) | 0.88 |
| CD25 |  |  |  |  |  |  |  |  |
|  | Logical Memory, Immediate Recall | 0.04 | (-0.09, 0.01) | 0.11 |  | 2.83 | (-0.69, 6.34) | 0.12 |
|  | Logical Memory, Delayed Recall | 0.06 | (-0.12, 0.01) | 0.09 |  | 4.05 | (-0.72, 8.82) | 0.10 |
|  | Paired associates learning | 0.01 | (-0.03, 0.06) | 0.63 |  | -0.76 | (-3.83, 2.31) | 0.63 |
|  | Digit Symbol Substitution Test | 0.15 | (-0.29, -0.01) | 0.03 |  | 9.95 | (0.32, 19.59) | 0.04 |
|  | Block Design | 0.03 | (-0.12, 0.05) | 0.47 |  | 1.39 | (-4.14, 6.92) | 0.62 |
| CD163 |  |  |  |  |  |  |  |  |
|  | Logical Memory, Immediate Recall | 0.01 | (-0.06, 0.04) | 0.64 |  | 0.61 | (-2.85, 4.07) | 0.73 |
|  | Logical Memory, Delayed Recall | 0.06 | (-0.11, 0.00) | 0.05 |  | 3.55 | (-0.47, 7.57) | 0.08 |
|  | Paired associates learning | 0.01 | (-0.05, 0.03) | 0.62 |  | 0.65 | (-2.20, 3.49) | 0.66 |
|  | Digit Symbol Substitution Test | 0.11 | (-0.25, 0.03) | 0.14 |  | 7.64 | (-2.07, 17.35) | 0.12 |
|  | Block Design | 0.04 | (-0.12, 0.04) | 0.34 |  | 2.30 | (-3.37, 7.97) | 0.43 |

Legend. Beta coefficients for the cognitive slope refer to the regression of cognitive slopes on biomarker levels. Beta coefficients for the cognitive level refer to the regression of cognitive level, or baseline intercept, on biomarker levels. Models are adjusted for age, sex, years of education, and vascular risk factors.

**Appendix e-1**

**Clinical and Cognitive Assessment of Participants**

The clinical and cognitive assessments were completed annually at both the NIH and JHU (for details, see Albert et al., 2014). The present study utilizes consensus diagnoses completed by the staff of the JHU BIOCARD Clinical Core. All cases were handled in a manner comparable with those employed in the National Institute on Aging Alzheimer's Disease Centers program. First a syndromic diagnosis was established, using three sources of information: (1) clinical data pertaining to the medical, neurological, and psychiatric status of the individual; (2) reports of changes in cognition by the individual and by collateral sources; and (3) decline in cognitive performance, based on review of longitudinal testing from multiple domains (and comparison to published norms), was established.

Second, if a subject was deemed to be impaired, the decision about the likely etiology of the syndrome was based on the medical, neurologic, and psychiatric information collected at each visit, as well as medical records obtained from the subject, where necessary. More than one etiology could be endorsed for each subject (e.g., AD and vascular disease). The consensus diagnosis procedures followed the diagnostic recommendations incorporated in the NIA/AA working group reports for the diagnosis of MCI (Albert *et al*., 2011) and dementia due to AD (McKhann *et al*., 2011). The diagnosis of Impaired Not MCI typically reflected contrasting information from the CDR interview and the cognitive test scores (i.e., the subject or collateral source expressed concerns about cognitive changes in daily life but the cognitive testing did not show changes, or vice versa). Diagnoses (and determination of likely etiology) were made without knowledge of the biomarker measures.

The estimated age of onset of clinical symptoms was established separately, based primarily on the semi-structured interview with the subject and the collateral source (the CDR). The age of symptom onset was established for the first visit at which the subject was deemed to be impaired and was reconfirmed on subsequent visits; thus, there is a single age of symptom onset for each subject with a diagnosis of MCI or dementia.

References

Albert,M., Soldan,A., Gottesman,R., McKhann,G., Sacktor,N., Farrington,L., Grega,M., Turner,R., Lu,Y., Li,S. et al 2014. Cognitive changes preceding clinical symptom onset of mild cognitive impairment and relationship to ApoE genotype. Curr. Alzheimer Res. 11:773-784.

Albert MS, DeKosky ST, Dickson D, Dubois B, Feldman HH, Fox NC, Gamst A, Holtzman DM, Jagust WJ, Petersen RC, Snyder PJ, Carrillo MC, Thies B, Phelps CH. The diagnosis of mild cognitive impairment due to Alzheimer's disease: recommendations from the National Institute on Aging-Alzheimer's Association workgroups on diagnostic guidelines for Alzheimer's disease. Alzheimers Dement. 2011 May;7(3):270-9. doi: 10.1016/j.jalz.2011.03.008.

McKhann GM, Knopman DS, Chertkow H, Hyman BT, Jack CR Jr, Kawas CH, Klunk WE, Koroshetz WJ, Manly JJ, Mayeux R, Mohs RC, Morris JC, Rossor MN, Scheltens P, Carrillo MC, Thies B, Weintraub S, Phelps CH. The diagnosis of dementia due to Alzheimer's disease: recommendations from the National Institute on Aging-Alzheimer's Association workgroups on diagnostic guidelines for Alzheimer's disease. Alzheimers Dement. 2011 May;7(3):263-9. doi: 10.1016/j.jalz.2011.03.005.
